# Visible Light-Driven Photocatalytic Performance of N-Doped ZnO/g-C_3_N_4_ Nanocomposites

**DOI:** 10.1186/s11671-017-2297-0

**Published:** 2017-09-06

**Authors:** Ji-Zhou Kong, Hai-Fa Zhai, Wei Zhang, Shan-Shan Wang, Xi-Rui Zhao, Min Li, Hui Li, Ai-Dong Li, Di Wu

**Affiliations:** 1 National Laboratory of Solid State Microstructures, Materials Science and Engineering Department, Nanjing University, Nanjing, 210093 China; 2 Jiangsu Precision and Micro-Manufacturing Technology Laboratory, College of Mechanical and Electrical Engineering, Nanjing University of Aeronautics and Astronautics, Nanjing, 210016 China; 3 Henan Key Laboratory of Photovoltaic Materials, College of Physics and Materials Science, Henan Normal University, Xinxiang, 453007 China

**Keywords:** N-doped ZnO, g-C_3_N_4_, Composite, Visible-light irradiation, Photocatalytic degradation

## Abstract

N-doped ZnO/g-C_3_N_4_ composites have been successfully prepared via a facile and cost-effective sol-gel method. The nanocomposites were systematically characterized by XRD, FE-SEM, HRTEM, FT-IR, XPS, and UV-vis DRS. The results indicated that compared with the pure N-doped ZnO, the absorption edge of binary N-doped ZnO/g-C_3_N_4_ shifted to a lower energy with increasing the visible-light absorption and improving the charge separation efficiency, which would enhance its photocatalytic activity. Compared with the pure g-C_3_N_4_, ZnO, N-doped ZnO and the composite ZnO/g-C_3_N_4_, the as-prepared N-doped ZnO/g-C_3_N_4_ exhibits a greatly enhanced photocatalytic degradation of methylene blue and phenol under visible-light irradiation. Meanwhile, N-doped ZnO/g-C_3_N_4_ possesses a high stability. Finally, a proposed mechanism for N-doped ZnO/g-C_3_N_4_ is also discussed. The improved photocatalysis can be attributed to the synergistic effect between N-doped ZnO and g-C_3_N_4_, including the energy band structure and enhanced charge separation efficiency.

## Background

Photocatalytic degradation of the organic compounds using solar energy as the energy source has attracted considerable interest for the environmental protection [1–3]. As is known, ZnO is one kind of the important semiconductor photocatalysts because of its unique advantages, such as its low price, high photocatalytic activity, and nontoxicity [4, 5]. However, the disadvantages such as low charge separation efficiency, susceptibility to photocorrosion, and poor visible light absorbance limited its widely commercial applications [6, 7]. Doping with the metal and/or nonmetal ions, coupling with other semiconductors, and surface sensitization with metal complexes could be considered as the feasible approaches to improve its utilization of solar energy and charge separation efficiency [7, 8]. It is reported that the nonmetal element N doping effectively improved the light absorption of ZnO in the visible range [9]. The nitrogen atom is closest in the atomic size and electronegativity to oxygen atom [10], so N doping could result in the minimum strain in ZnO. Regrettably, that N-doped ZnO does not exhibit excellent photocatalytic efficiency unlike N-doped TiO_2_ [11].

Graphitic carbon nitride (g-C_3_N_4_) is a relatively novel, versatile, and promising metal-free polymeric semiconductor photocatalyst [12–14], due to its special semiconducting properties and low cost. It has been widely investigated for its great potential in degrading environmental pollutants [12], catalyzing water splitting for H_2_ evolution [13], and reducing carbon dioxide [14] under irradiation. However, the easy recombination of its photogenerated charges restricts its photocatalytic performance and greatly limits its wide practical application [15]. Constructing a suitable heterojunctional composite is one of the most general methods to improve the photogenerated charge separation [16–18]. Coupling g-C_3_N_4_ with ZnO could yield an excellent heterostructure, since these two semiconductors have well-matched, overlapping band structures [6]. Under visible light irradiation, the initial electron excited from the valence band (VB) can transfer to the conduction band (CB) of the g-C_3_N_4_, then further transfer to the CB of ZnO [6, 8, 19], resulting in an improved photocatalytic activity of ZnO/g-C_3_N_4_. Recently, Shanker et al. [10] reported that N-doped ZnO/g-C_3_N_4_ hybrid core–shell shows a greatly enhanced visible-light photocatalysis for the degradation of rhodamine B. However, to the best of our knowledge, there are no works reported about N-doped ZnO/g-C_3_N_4_ for the visible-light degradation of the volatile organic pollutants such as phenol and methylene blue (MB) until now.

In this work, the N-doped ZnO/g-C_3_N_4_ composite photocatalysts were synthesized via the sol-gel method. The as-prepared composite exhibited significantly enhanced photocatalytic degradation of MB and phenol under visible-light irradiation. Finally, the possible mechanism about the photocatalytic degradation of MB and phenol was also investigated.

## Methods

### Preparation of N-Doped ZnO/g-C_3_N_4_ Nanocomposites

g-C_3_N_4_ powder was prepared by heating melamine [20]. In brief, 5 g of melamine was placed in an alumina crucible with a cover that was firstly heated to 80 °C, followed by calcining at 550 °C for 4 h in a muffle furnace. After natural cooling to room temperature, the obtained sample was milled into powder. Then, g-C_3_N_4_ powder was ultrasonicated in water and centrifuged to remove the unexfoliated g-C_3_N_4_.

In order to prepare the sol of N-doped ZnO, the equal mole of zinc acetate and urea were dissolved in ethanol [10, 21]. An appropriate amount of g-C_3_N_4_ was added to the above solution with continuous stirring. The solution was then kept at 80 °C water bath for 5 h. After that, the resultant mixture was dried and heated at 400 °C for 1 h to obtain N-doped ZnO/g-C_3_N_4_ loaded with 50 mol% N-doped ZnO, which is marked as N-ZnO/g-C_3_N_4_.

The sol for synthesizing ZnO was prepared by using above method without adding any urea. Then, the ZnO/g-C_3_N_4_ nanocomposites loaded with 50 mol% ZnO were prepared under the same experimental conditions, except for calcining in air.

### Characterization

X-ray diffraction (XRD) patterns were carried out on a Rigaku D/max 2000 diffractometer employing Cu K*α* radiation. The morphology and microstructure of the samples were examined by a field-emission scanning electron microscopy (FE-SEM; Ultra 55, Zeiss) and high-resolution transmission electron microscopy (HRTEM; Tecnai G^2^ F20 S-Twin, FEI). Fourier transform infrared (FT-IR) spectra were recorded on a Nicolet Nexus-870 infrared spectroscopy (Thermo Nicolet) in the range of 400–4000 cm^−1^ using KBr pellets. Chemical compositions of the particle samples were analyzed by an X-ray photoelectron spectroscopy (XPS; Thermo Fisher K-Alpha) with Al K*α* radiation, and all the spectra were calibrated by assigning the peak at 284.6 eV. The Brunauer–Emmett–Teller (BET) surface area was estimated by a surface area apparatus (TriStar-3000, Micromeritics). UV-vis diffuse reflectance spectra (UV-vis DRS) were recorded by a UV-vis spectrophotometer (UV-3600, Shimadzu) equipped with an integrating sphere in the range of 200–800 nm, and BaSO_4_ was used as a reference.

### Photocatalytic Activity

The photocatalytic activity of the as-prepared photocatalysts was evaluated via the degradation of MB and phenol in aqueous solution. A solar simulator (300 W Xe lamp) with a 420 nm cutoff filter provides the visible-light irradiation with the light intensity of 120 mW/cm^2^. The catalyst (0.5 g/L for MB and 5 g/L for phenol) and 100 ml of aqueous solution containing 10 mg/L MB (or 5 mg/L phenol) were placed in a glass reactor with continuous stirring at 250 rpm. Prior to irradiation, the pollutant solutions suspended with photocatalysts were stirred in the absence of light for 30 min to attain the equilibrium adsorption/desorption between photocatalyst powders and MB/phenol. During the reaction, the temperature was maintained at 25 ± 1 °C. For each given irradiation time, about 3 mL of the reacted solution was withdrawn and centrifuged at 12,000 rpm for 30 min to remove the photocatalyst. Then, the concentration of the centrifuged solution was determined by a UV-vis-NIR spectrophotometer (UV-3600, Shimadzu) with the maximum absorption of MB and phenol at 664 m and 270 nm, respectively). After visible-light irradiation, the photocatalysts were collected, washed, and dried at 100 °C for 12 h. The stability of the photocatalysts was checked by running four separate cycles. The total organic carbon (TOC) was determined using a Shimadzu TOC-2000 analyzer. For exploring the active species during the photocatalytic reaction, the effect of various scavengers on the degradation of dye was investigated. The method was similar to the former photocatalytic activity test under visible-light irradiation.

## Results and Discussion

**Fig. 1:**
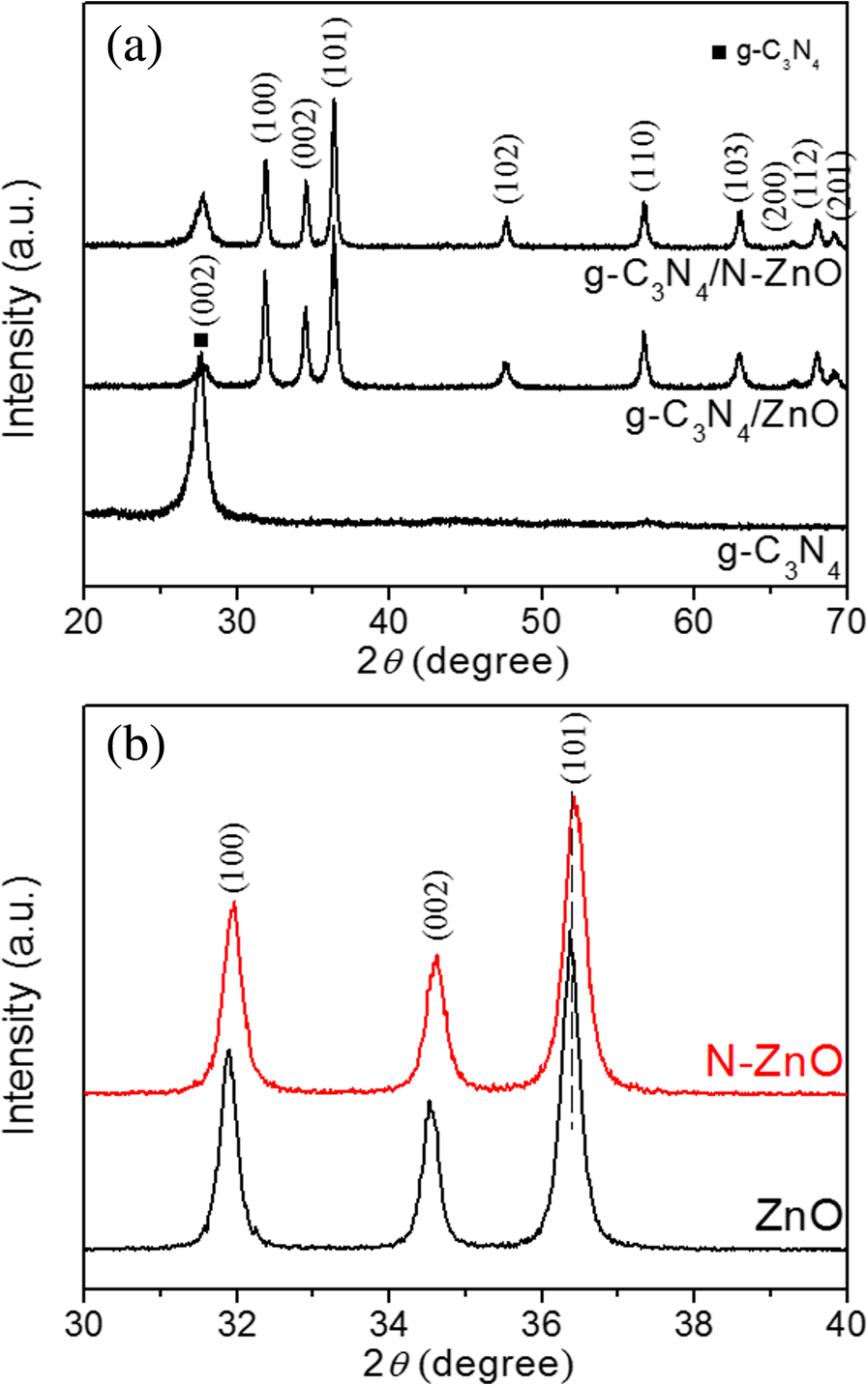
XRD patterns (**a**) of g-C_3_N_4_, ZnO/g-C_3_N_4_ and N-ZnO/g-C_3_N_4_ and the Bragg angle shift (**b**) of ZnO and N-ZnO in corresponding composites

Figure 1 shows XRD patterns of g-C_3_N_4_, ZnO, N-ZnO ZnO/g-C_3_N_4_, and N-ZnO/g-C_3_N_4_ composites. The main characteristic peaks can be indexed as the hexagonal ZnO with wurtzite structure (JPCDS 36-1451). A strong peak at 27.5°, corresponding to the characteristic diffraction peak (002) of g-C_3_N_4_ [10, 22], can be also observed. Hence, we can conclude that the introduction of nitrogen does not change the crystal structure of ZnO. Moreover, as presented in Fig. 1b, the diffraction peaks for N-ZnO in N-ZnO/g-C_3_N_4_ have a slight red shift, as compared with those for ZnO in ZnO/g-C_3_N_4_, indicating an overall contraction of the lattice parameters [10]. The crystallite size of N-ZnO (38.6 nm) derived from the Scherer formula is smaller than that of ZnO (45.8 nm). This may be ascribed to the N doping which can inhibit the growth of ZnO [21]. After doping with N, the diffraction peak is obviously broader than that of ZnO (Fig. 1b), due to its low crystallinity that resulted from the introduction of N into the crystal lattice of ZnO. The values of specific surface area are 15.3 and 18.5 m^2^/g for ZnO/g-C_3_N_4_ and N-ZnO/g-C_3_N_4_, respectively. By comparison of the pure ZnO and N-ZnO, the BET surface areas of the composites are greatly increased. The increase of BET surface area indicates that the separation and migration efficiency of the photogenerated carriers would be improved, which could be in favor to the photocatalytic activity of composite.

**Fig. 2:**
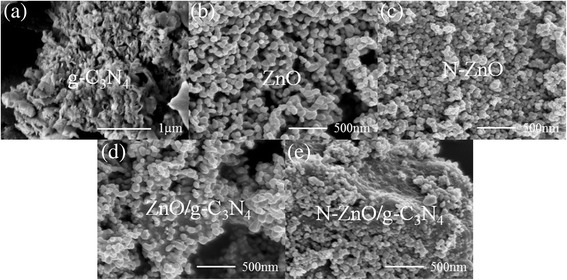
SEM images of **a** pure g-C_3_N_4_, **b** ZnO, **c** N-ZnO, **d** ZnO/g-C_3_N_4_, and **e** N-ZnO/g-C_3_N_4_

The morphology and microstructure of as-prepared photocatalysts were analyzed by TEM and SEM. As shown in Fig. 2a, pure g-C_3_N_4_ exhibits the sheet-like morphology with the fluffy structure. Figure 2b, c shows the SEM images of ZnO and N-doped ZnO samples, respectively. As compared with pure ZnO, the N-doped ZnO shows smaller crystallite size with a relatively uniform diameter, which is in agreement with the results calculated using the Scherrer formula based on the XRD data. Moreover, the morphologies of ZnO/g-C_3_N_4_ and N-ZnO/g-C_3_N_4_ composite photocatalysts are evidently different from those of g-C_3_N_4_. Obviously, the ZnO and N-ZnO nanoparticles in ZnO/g-C_3_N_4_ and N-ZnO/g-C_3_N_4_ are dispersed over the composite surface, respectively (Fig. 2d, e). The uniform distribution of nanoparticles on g-C_3_N_4_ could minimize the aggregation of ZnO and N-ZnO, and maximize the reactive sites, which could be in favor of the photocatalytic reactions [7]. Unlike ZnO/g-C_3_N_4_, it can be found that the surface of N-ZnO/g-C_3_N_4_ is obviously rough, and it can be attributed to the N-ZnO particles which have been assembled uniformly on the surface of N-ZnO/g-C_3_N_4_ during the heating treatment.

**Fig. 3:**
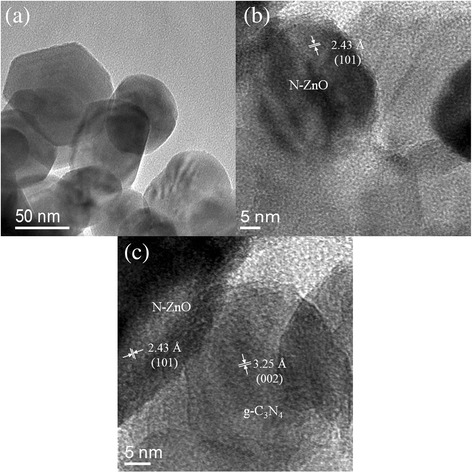
TEM images of N-ZnO/g-C_3_N_4_

The corresponding TEM images in Fig. 3 likewise indicate that the properly heterostructured composite exists, where N-ZnO nanospheres on the surface of N-ZnO/g-C_3_N_4_ are well attached to the g-C_3_N_4_. Figure 3b, c shows the HRTEM images of N-ZnO/g-C_3_N_4_. The measured lattice-fringes spacing of 3.25 and 2.43 Å are in well agreement with the crystal planes (002) and (101) of g-C_3_N_4_ and N-ZnO, respectively.

**Fig. 4:**
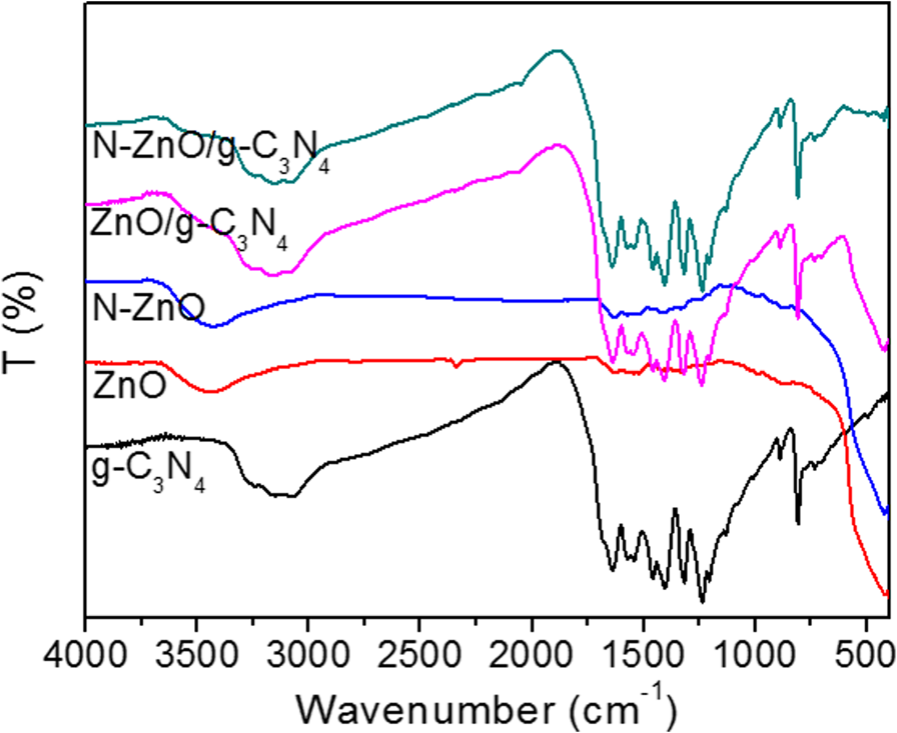
FT-IR spectra of g-C_3_N_4_, ZnO, N-ZnO, ZnO/g-C_3_N_4_, and N-ZnO/g-C_3_N_4_

Fourier transform infrared (FT-IR) spectra of the g-C_3_N_4_, ZnO, N-ZnO, and the composite materials are shown in Fig. 4. For ZnO and N-doped ZnO, the peaks in the region from 400 to 560 cm^−1^ is corresponding to the bending vibrations of Zn–O bands [6, 10], which were observed in all of the samples except for g-C_3_N_4_. In the spectrum of g-C_3_N_4_, the peaks at 1243 and 1637 cm^−1^ correspond to the stretching vibrations of C−N and C=N, respectively [10]. The peaks at 810 cm^−1^ originate from the breathing mode of the s-triazine ring units [23, 24]. The broad absorption band at a high wave number around 3100–3400 cm^−1^ is attributed to the stretching vibration of N−H bonds in the −NH_2_ and/or =N−H amines, as well as the hydroxyl groups of the chemisorbed and/or physisorbed H_2_O molecules [10, 23]. It can be clearly seen that the main characteristic IR peaks of g-C_3_N_4_ exist in the ZnO/g-C_3_N_4_ and N-ZnO/g-C_3_N_4_ composites, suggesting that the structural features of g-C_3_N_4_ are maintained after the hybridization process, in good agreement with the XRD results. Additionally, the main characteristic peaks of g-C_3_N_4_ in the composites slightly shift to a high wave number. This red shift could be attributed to the fact that the extendedly conjugated system appears in the heterostructured composites [10, 25].

**Fig. 5:**
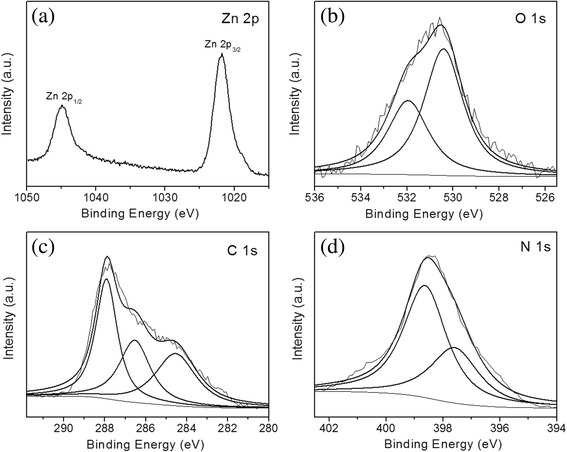
XPS spectra of N 1s for N-ZnO/g-C_3_N_4_: **a** Zn 2p, **b** O 1s, **c** C 1s, and **d** N 1s

Figure 5 shows XPS spectra of N-ZnO/g-C_3_N_4_. Two peaks at 1021.8 and 1044.9 eV in Fig. 5a are attributed to Zn 2p_3/2_ and 2p_1/2_, respectively. The O 1s peak is fitted with the non-linear least square fit program using Gauss–Lorentzian peak shapes. After deconvolution, there are two fitted peaks located at 530.4 and 532.0 eV. The peak at 530.4 eV can be assigned to the O^2−^ ions in ZnO [26]. The other peak of 532.0 eV can be attributed to the chemically absorptive oxygen and/or hydroxyl group [26] on the surface of the composite photocatalyst. The C1s spectrum (Fig. 5c) of N-ZnO/g-C_3_N_4_ can also be fitted into three peaks, corresponding to three basic kinds of C states. The binding energy of 284.6 eV is attributed to the adventitious carbon (C−C) on the surface of N-doped ZnO/g-C_3_N_4_ [3, 6]. The C1s peaks at 286.5 and 287.8 eV are assigned to the sp^3^- and sp^2^-bonded carbon in N−C=N of the composite, respectively. With regard to the N1s spectrum of g-C_3_N_4_, there are three peaks after deconvolution, indicating three different kinds of N states [27, 28], the pyridinic N (C−N−C) at 398.5 eV, pyrrolic N (N−[C]_3_) at 399.8 eV, and graphitic N (C−NH) at 401.0 eV. Above the three kinds of N states are the basic units of g-C_3_N_4_. In the N1s spectrum (Fig. 5d), the peaks at binding energies of 397.5 and 398.6 eV can be assigned to the anionic N in O−Zn−N linkage [29] and sp^2^-hybridized N [28]. Here, we can conclude that the framework of g-C_3_N_4_ is not changed even if it has been combined with N-ZnO particles. Additionally, the results of XRD, FT-IR, and XPS confirm that there are both N-doped ZnO and g-C_3_N_4_ species in the heterojunction structure.

**Fig. 6:**
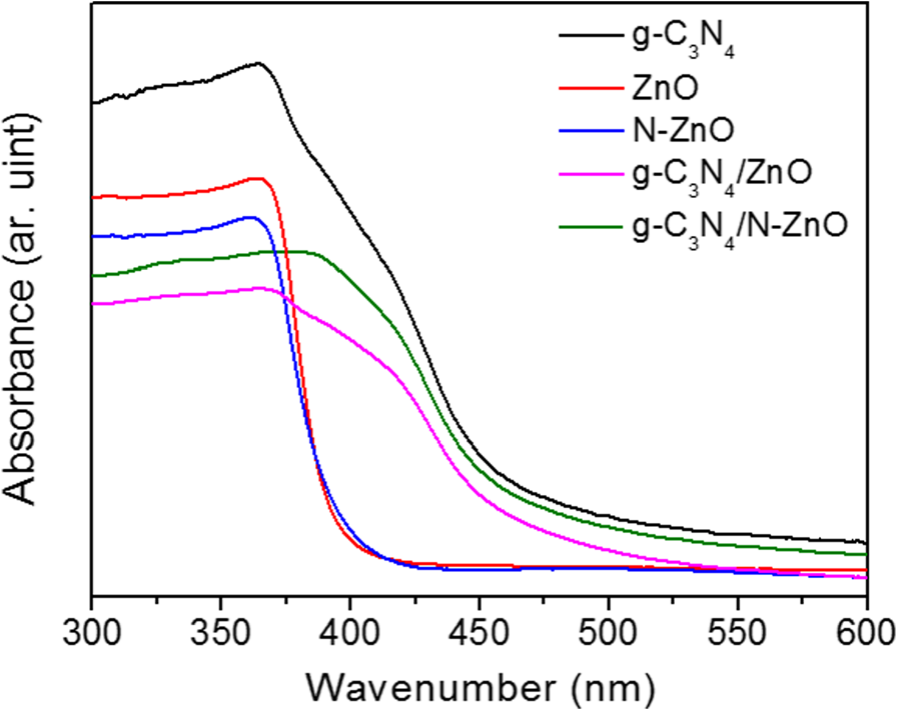
UV-visible absorption spectra of g-C_3_N_4_, ZnO, N-ZnO, ZnO/g-C_3_N_4_, and N-ZnO/g-C_3_N_4_

The UV-visible diffuse reflectance spectra of the prepared powder samples were also measured with a pressed BaSO_4_ as a reference. The resulting data is plotted as the remission function shown in Eq. 1.

1 $$ F(R)=\raisebox{1ex}{${\left(1-R\right)}^2$}\!\left/ \!\raisebox{-1ex}{$2R$}\right. $$

where *R* is the diffuse reflectance based on the Kubelka–Monk theory. The band gap energies (*E*_g_) of the direct bandgap semiconductor were estimated from the Eq. 2 by extrapolating the linear part.

2 $$ {\left(F(R)\cdot hv\right)}^2=A\left( hv-{E}_g\right) $$

where *A* is the absorption constants decided by the direct bandgap semiconductor of pure g-C_3_N_4_, ZnO, N-ZnO, the nanocomposite ZnO/g-C_3_N_4_, and N-ZnO/g-C_3_N_4_. Figure 6 shows the UV-vis absorption spectra of the as-prepared samples. It can be obviously seen that the absorption edge of the pure g-C_3_N_4_ is around 470 nm [6], corresponding to a band gap of 2.63 eV. As shown in Fig. 6, ZnO has a clear absorption edge around 390 nm in the UV range. Compared with the pure ZnO, an obvious red shift of the absorption edge towards higher a wavelength is detected in the N-ZnO sample, due to the contribution of nitrogen to the top of the valence band (VB) of ZnO which can drive the absorption of N-doped ZnO close to the visible region [10]. Thus, the band gap of ZnO is reduced from 3.21 to 3.10 eV after nitrogen doping. Another significant change is the enhanced absorption in the visible-light region ranged from 400 to 600 nm for the ZnO/g-C_3_N_4_ and N-ZnO/g-C_3_N_4_, compared with the pure ZnO and N-ZnO. It can be attributed to the effective surface hybridization [6, 30] between g-C_3_N_4_ and ZnO (N-doped ZnO) on its surface. Further, N-ZnO/g-C_3_N_4_ (2.73 eV) show the broader absorption edge in the visible region, as compared with ZnO/g-C_3_N_4_ (2.85 eV), which is in favor of the photodegradation of dyes under visible-light irradiation.

The conduction band (CB) and valence band (VB) edges of g-C_3_N_4_ and ZnO locate approximately at − 1.3 eV/+ 1.4 eV and − 0.5 eV/+ 2.7 eV vs. NHE [6, 8, 31], respectively. For N-ZnO, the edge potentials of VB and CB can be determined by using the following equation [32].

3 $$ {E}_{\mathrm{VB}}=X-{E}_{\mathrm{e}}+0.5{E}_{\mathrm{g}} $$

4 $$ {E}_{\mathrm{CB}}={E}_{\mathrm{VB}}-{E}_{\mathrm{g}} $$

where *E*_VB_, *X*, and *E*_e_ are the edge potential of the valence band, the absolute electronegativity of the semiconductor which is determined by the geometric mean of the electronegativity of the constituent atoms, and the energy of the free electron on the hydrogen scale (~ 4.5 eV), respectively [10, 24]. The *E*_VB_ and *E*_CB_ of N-doped ZnO are calculated to be 2.65 and − 0.45 eV, respectively.

**Fig. 7:**
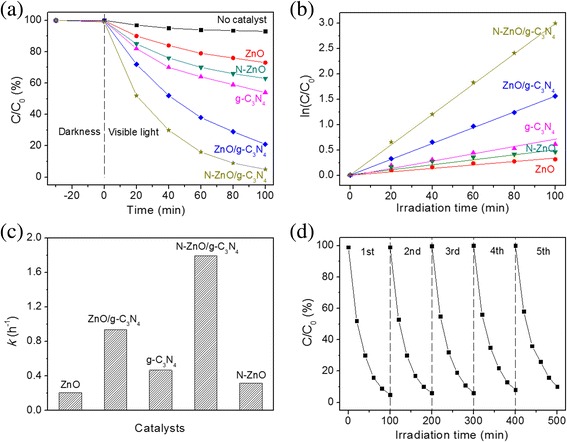
**a** Photocatalytic degradation of MB by using ZnO, N-ZnO, g-C_3_N_4_, ZnO/g-C_3_N_4_, and N-ZnO/g-C_3_N_4_ catalysts under visible-light irradiation, **b** the corresponding ln (*C*/*C*_0_) vs. time curves, **c** the rate constants of MB photodegradation, and **d** five cycles of MB degradation for N-ZnO/g-C_3_N_4_

Figure 7 shows the photocatalytic degradation of MB by using the prepared photocatalysts under the visible-light irradiation. Each sample performs a low adsorption capacity of MB. Almost no degradation of MB is observed in the absence of visible light or catalyst, demonstrating that MB is stable under the above conditions. As shown in Fig. 7a, after N doping, the photocatalytic stability of N-ZnO is improved, indicating that the introduction of N doping can suppress the recombination of photogenerated charge carriers. Meanwhile, the photocatalytic activities of ZnO/g-C_3_N_4_ and N-ZnO/g-C_3_N_4_ composite catalysts are obviously higher than those of the pure reference samples. The improved photocatalytic activity is attributed to the heterostructure of composites, which can promote the photogenerated electron transfer and suppress the recombination of the electron–hole pairs [7, 33]. Moreover, the N-ZnO/g-C_3_N_4_ catalyst exhibit higher photocatalytic activity than ZnO/g-C_3_N_4_, in spite of ZnO/g-C_3_N_4_ heterostructure. It can be owed to its improved absorption in the visible region to produce the electron–hole pairs and narrower band gap energy. The experimental results were fitted to the pseudo-first-order kinetics. At low initial pollutant concentration, the constant rate *k* was given by Eq. 5.

5 $$ \ln \raisebox{1ex}{$C$}\!\left/ \!\raisebox{-1ex}{${C}_0$}\right.=- kt $$

Here, *k* and *t* represent the first-order rate constant (h^−1^) and the irradiation time (h), respectively. *C*_0_ is the initial concentration of MB, and *C* is the concentration at a reaction time of *t*. The corresponding plots of ln (*C*_0_/*C*) vs. the irradiation time for photodegradation of MB are shown in Fig. 7b. A linear relation between ln (*C*_0_/*C*) and the irradiation time has verified that the photodegradation of MB follows the first-order kinetics. The calculated first-order rate constants (*k*) are presented in Fig. 7c. The kinetic constant of N-ZnO/g-C_3_N_4_ is 1.794 h^−1^, which is 5.68, 3.85, and 1.91 times higher than those of N-ZnO (0.316 h^−1^), g-C_3_N_4_ (0.466 h^−1^), and ZnO/g-C_3_N_4_ (0.937 h^−1^). Apparently, N-ZnO/g-C_3_N_4_ exhibits the highest degradation efficiency of MB among all of the catalysts. In order to evaluate the stability of photocatalyst, the recyclic experiments about the photodegradated MB are performed with the N-ZnO/g-C_3_N_4_ catalyst. As shown in Fig. 7d, the photocatalytic activity of N-ZnO/g-C_3_N_4_ exhibits an extremely limited decline. The degradation efficiency of MB solution is nearly 90% after 100 min even at the fifth recycling experiment.

**Fig. 8:**
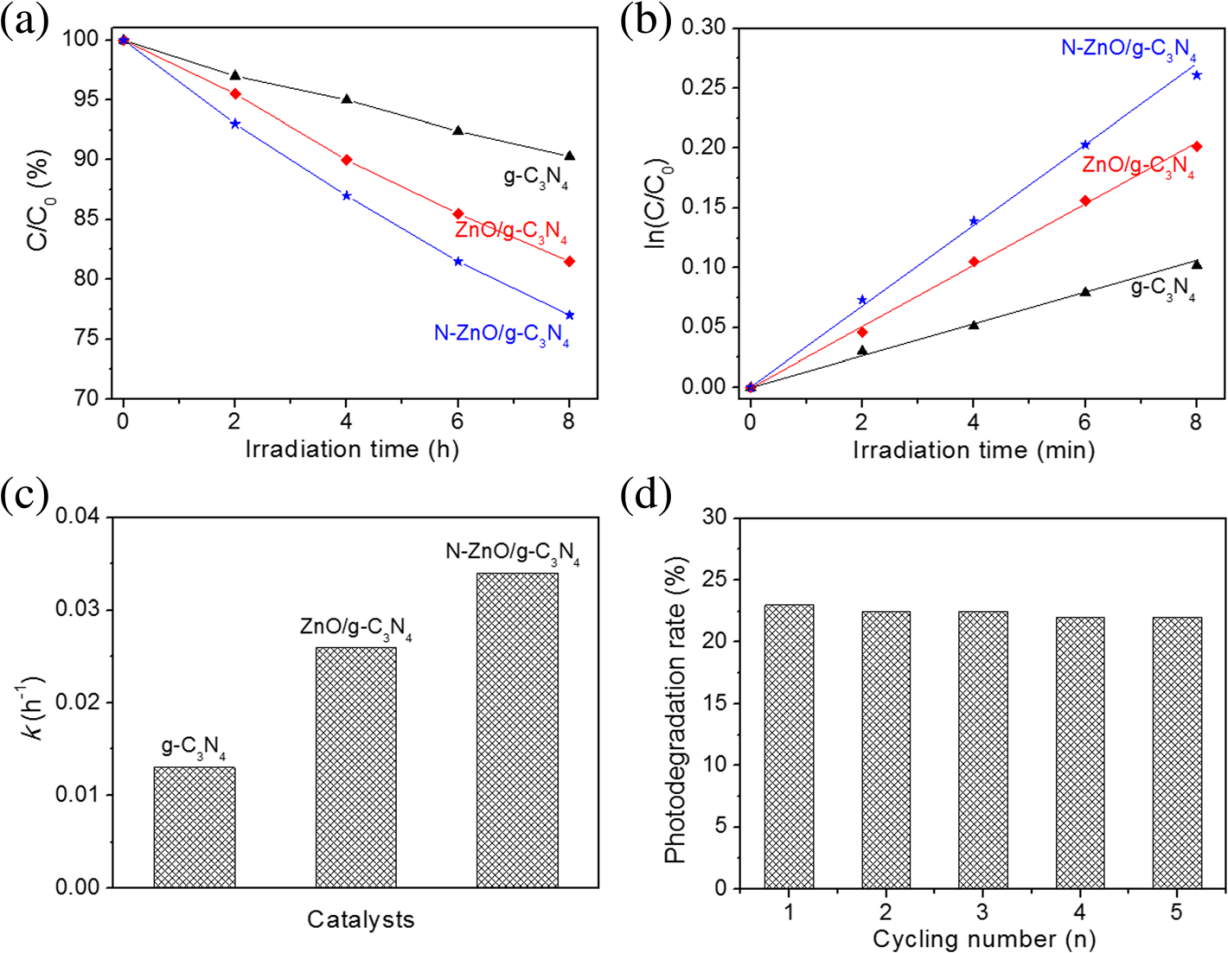
**a** Photocatalytic degradation of phenol by using g-C_3_N_4_, ZnO/g-C_3_N_4_ and N-ZnO/g-C_3_N_4_ catalysts under visible-light irradiation, **b** the corresponding first-order kinetic plots and **c** the rate constants of phenol degradation, and **d** degradation efficiency after 8 h for five repeated processes by the N-ZnO/g-C_3_N_4_ photocatalyst

Phenol was also adopted as a representative recalcitrant pollutant to evaluate the photocatalytic performance of catalysts under visible-light irradiation, and the results are showed in Fig. 8. The photocatalytic degradation rates are expressed by Eq. 5, where *C* is the temporal concentration of phenol after irradiation, and *C*_0_ is the concentration after adsorption process. Among all of the catalysts, N-ZnO/g-C_3_N_4_ presents the highest photodegradation efficiency of phenol. Based on the corresponding plots (Fig. 8b) of ln (*C*_0_/*C*) vs. the irradiation time, the kinetic constants of g-C_3_N_4_, ZnO/g-C_3_N_4_, and N-ZnO/g-C_3_N_4_ are 0.013, 0.026, and 0.034 h^−1^, respectively. More importantly, N-ZnO/g-C_3_N_4_ exhibits excellent cycling stability (Fig. 8d) for phenol removal. The above results further confirm that the N-ZnO/g-C_3_N_4_ catalyst performs excellent photodegradation ability of both MB and phenol, because of its smaller band gap and broader absorption edge in the visible region compared with that of ZnO/g-C_3_N_4_.

**Fig. 9:**
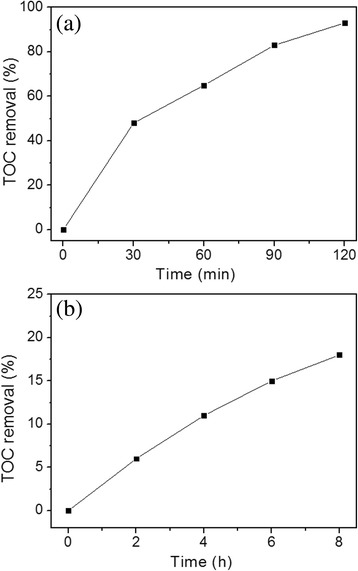
TOC removal of **a** MB and **b** phenol degradation by N-ZnO/g-C_3_N_4_ photocatalyst

By measuring the total organic carbon (TOC), the photocatalytic mineralization of the N-ZnO/g-C_3_N_4_ was carried out by degradation of MB and phenol in an aqueous solution under visible light irradiation. Figure 9 shows the TOC removal efficiency of MB and phenol as a function of reaction time. As shown in Fig. 9a, MB was degraded completely after 120 min, and the TOC removal rate reaches 93%. During the photodegradation process, MB was degraded to several intermediates, which may be the cleavage of one or more methyl groups substituent on the amine groups [34], and finally completely degraded to CO_2_ and H_2_O. By comparison, the mineralization of phenol only reached 18%. The main intermediates include hydroquinone (HQ), p-benzoquinone (p-BQ), 1, 3-dihydroxybenzene (DB), maleic anhydride (MA), and other low fatty acids (LFAs) [35, 36]. HQ and p-BQ can be easily oxidized into other intermediates or inorganic carbon. MA and LFA in the reaction system are difficult to be further oxidated and mineralized.

**Fig. 10:**
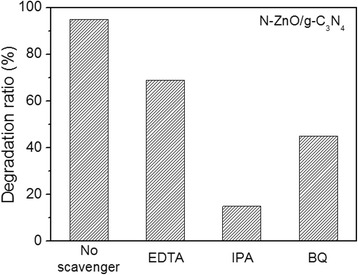
Effect of various scavengers on the degradation of MB over the N-ZnO/g-C_3_N_4_ catalyst

In the photocatalytic degradation of organic pollutants, there are series of photogenerated reactive species such as hole (h^+^), hydroxyl radicals (•OH), and superoxide anions (O_2_^•−^). In order to understand how the different reactive species play a role in the photodegradation process, the different scavengers were used to detect the active species during the photocatalytic degradation process. Here, EDTA, isopropyl alcohol (IPA), and benzoquinone (BQ) were adopted as hole (h^+^), hydroxyl radical (•OH), and superoxide anion (O_2_^•−^) scavengers at a concentration of 1.0 mM, respectively. Through the photocatalytic experiments, the N-ZnO/g-C_3_N_4_ composite exhibited the best degradation for the MB solution. So, the MB solution was chosen for the scavenging experiments. As shown in Fig. 10, an obvious decrease in the photocatalytic activity was also observed by the addition of EDTA and BQ, respectively, suggesting that both h^+^ and O_2_^•−^ play an important role in the photocatalytic reaction. Meanwhile, the photocatalytic degradation of MB was greatly suppressed by the addition of IPA, indicating that the hydroxyl radicals (•OH) are the main active species and play a dominant role in the photocatalytic reaction.

**Fig. 11:**
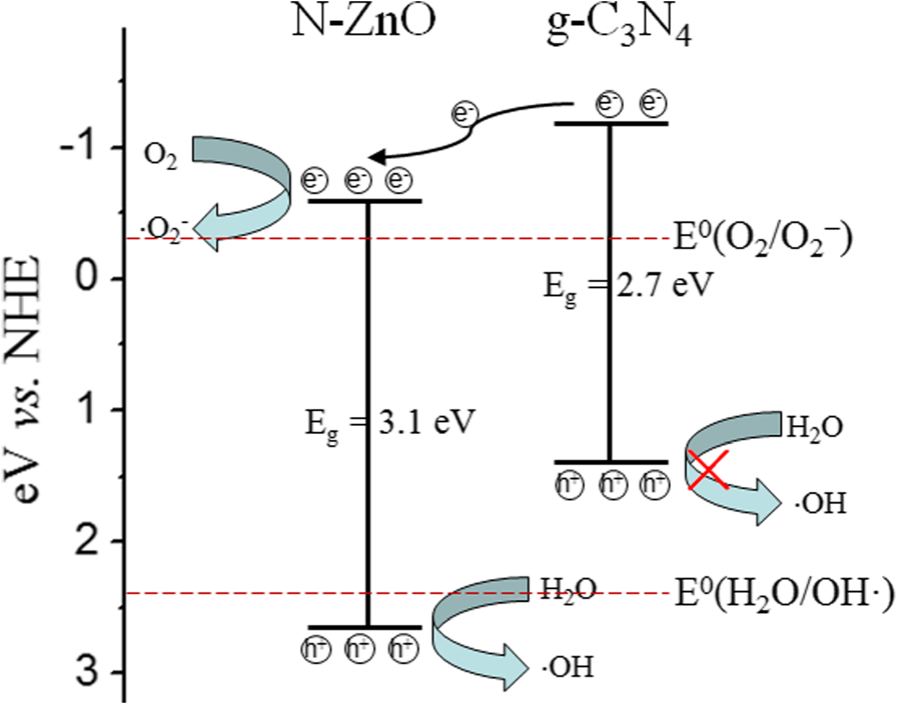
Schematic illustration of the band edge positions and proposed photocatalytic mechanism for the N-ZnO/g-C_3_N_4_ composite catalyst

The schematic illustration of the charge transfer and photocatalytic mechanism for the N-ZnO/g-C_3_N_4_ composite photocatalyst is shown in Fig. 11. Under the visible-light irradiation, the electron–hole pairs in the g-C_3_N_4_ and N-ZnO forms. And then, the excited-state electrons transport from the VB to the CB. Thus, the conduction-band electron and the valence-band hole separate on the surface of the catalyst. The photogenerated electrons transfer from the CB of g-C_3_N_4_ to the CB of N-ZnO due to the CB potential of g-C_3_N_4_ that is more negative than the CB edge of N-ZnO, so the separation efficiency of the electron–hole pair is enhanced [10]. The CB potential of N-doped ZnO (−0.45 eV vs. NHE) is below the standard redox potential *E* ^0^(O_2_/O_2_^−^) (−0.33 eV vs. NHE). So the photogenerated electrons in the CB of N-doped ZnO would subsequently react with the dissolved O_2_ to form the high oxidative hydroxyl radicals, which could oxidize the pollutants [5]. In addition, the photo-induced electrons have more negative potential to reduce the molecular oxygen to yield superoxide anion (O_2_^•−^), which then induces the degradation of organic pollutants. According to the previous reports [6, 30], the photo-excited holes on the VB of N-ZnO could transfer to the VB of g-C_3_N_4_. However, the photo-induced holes of g-C_3_N_4_ cannot oxidize the adsorbed H_2_O molecules to yield the hydroxyl radicals because the VB potential of g-C_3_N_4_ (+1.4 eV vs. NHE) is smaller than the standard redox potential E^0^(H_2_O/OH·) (+2.4 eV vs. NHE) [7, 10]. Based on the above discussion, the majority holes at the VB of N-ZnO do not transfer to the VB of g-C_3_N_4_. These photo-excited holes can directly oxidate the organic dye to obtain the reactive intermediates [5] and/or react with the H_2_O to form the hydroxyl radicals [2], which are the main reactive species in the photocatalytic reaction. Therefore, we propose that the N-ZnO/g-C_3_N_4_ heterojunction structure can enhance the separation of electron–hole pairs and reduce the recombination of charge carriers, leading to the increase of the photodegradation process.

## Conclusions

In summary, N-ZnO/g-C_3_N_4_ composite photocatalyst was successfully prepared via a facile sol-gel method. The addition of g-C_3_N_4_ enhances the light absorption in the visible region, generates more charge carriers, and simultaneously promotes the electron and hole segregation and migration. As compared with ZnO/g-C_3_N_4_, the N-ZnO/g-C_3_N_4_ shows higher photocatalytic activity on the degradation of MB and phenol, due to its improved absorption in the visible region and narrower band gap energy. The mechanism of the photocatalysis is analyzed, and the stability is also evaluated by recycling photocatalytic ability.
